# ACUTE PANCREATITIS GRAVITY PREDICTIVE FACTORS: WHICH AND WHEN TO USE THEM?

**DOI:** 10.1590/S0102-67202015000300016

**Published:** 2015

**Authors:** Alexandre de Figueiredo FERREIRA, Janaina Alves BARTELEGA, Hugo Corrêa de Andrade URBANO, Iure Kalinine Ferraz de SOUZA

**Affiliations:** 1 Faculty of Medicine, Federal University of Ouro Preto, Ouro Preto; 2 Faculty of Medicine, University José do Rosário Vellano, Belo Horizonte, MG, Brazil

**Keywords:** Acute pancreatitis, Prognosis, Disease severity index

## Abstract

**Introduction:**

Acute pancreatitis has as its main causes lithiasic biliary disease and alcohol abuse. Most of the time, the disease shows a self-limiting course, with a rapid recovery, only with supportive treatment. However, in a significant percentage of cases, it runs with important local and systemic complications associated with high mortality rates.

**Aim:**

To present the current state of the use of these prognostic factors (predictive scores) of gravity, as the time of application, complexity and specificity.

**Method:**

A non-systematic literature review through 28 papers, with emphasis on 13 articles published in indexed journals between 2008 and 2013 using Lilacs, Medline, Pubmed.

**Results:**

Several clinical, laboratory analysis, molecular and image variables can predict the development of severe acute pancreatitis. Some of them by themselves can be determinant to the progression of the disease to a more severe form, such as obesity, hematocrit, age and smoking. Hematocrit with a value lower than 44% and serum urea lower than 20 mg/dl, both at admission, appear as risk factors for pancreatic necrosis. But the PCR differentiates mild cases of serious ones in the first 24 h. Multifactorial scores measured on admission and during the first 48 h of hospitalization have been used in intensive care units, being the most ones used: Ranson, Apache II, Glasgow, Iget and Saps II.

**Conclusion:**

Acute pancreatitis is a disease in which several prognostic factors are employed being useful in predicting mortality and on the development of the severe form. It is suggested that the association of a multifactorial score, especially the Saps II associated with Iget, may increase the prognosis accuracy. However, the professional's preferences, the experience on the service as well as the available tools, are factors that have determined the choice of the most suitable predictive score.

## Abstract (pt)

**Introdução:**

A pancreatite aguda tem como principais causas a doença biliar litiásica e a ingestão abusiva de álcool. Na maioria das vezes, ela apresenta curso autolimitado, com rápida recuperação somente com tratamento suportivo. No entanto, significativo percentual de casos cursa com complicações locais e sistêmicas com elevados índices de mortalidade.

**Objetivo:**

Apresentar o estado atual da utilização dos fatores (escores) prognósticos (preditivos) de gravidade, quanto ao momento da aplicação, complexidade e especificidade.

**Método:**

Revisão não sistemática da literatura através do estudo de 28 trabalhos, com ênfase em 13 artigos publicados em periódicos indexados no período de 2008 a 2013. As bases de dados utilizadas foram: Lilacs, Medline e Pubmed.

**Resultados:**

Diversas variáveis clínico-laboratoriais, moleculares e de imagem podem predizer o desenvolvimento da pancreatite aguda grave. Algumas isoladamente são determinantes da evolução da doença para a forma mais grave, tais como: obesidade, hematócrito, idade e tabagismo. Hematócrito com valor >44% e ureia sérica >20 mg/dl na admissão configuram-se como fatores de risco para a necrose pancreática. Já a PCR diferencia os casos leves dos graves nas primeiras 24 h. Escores multifatoriais mensurados à admissão e durante as primeiras 48 h de internação têm sido utilizados em serviços de terapia intensiva, sendo os mais empregados: Ranson, Apache II, Glasgow, Iget e Saps II.

**Conclusão:**

A pancreatite aguda é doença em que se empregam diversos fatores prognósticos, úteis na predição da mortalidade e do desenvolvimento da forma grave. Sugere-se que a associação de um escore multifatorial, em especial o Saps II associado ao Iget, permite aumento da acurácia da predição prognóstica. Todavia, as preferências do profissional, a experiência do serviço, bem como, as ferramentas disponíveis, são fatores que têm determinado a escolha do escore preditor mais adequado.

## INTRODUCTION

Acute pancreatitis is a disease triggered by the abnormal activation of pancreatic enzymes and the release of a number of inflammatory mediators, whose etiology corresponds in about 80% of the cases to lithiasic biliary disease or excessive alcohol intake. Diagnosis is done by clinical, laboratory or image findings. Most of the time, it is self-limited to the pancreas and with minimal systemic effects. This mild form is characterized by presenting good clinical outcome and lower mortality rates. However, approximately 10-20% of the cases, the clinical course is more intense and with extensive systemic effects, leading to up 40% mortality. The correct diagnosis, established early and determining its severity factors, are of fundamental importance to the proper therapeutic management 1 .

After the Atlanta Symposium (1992), came to be accepted two clinical presentations of well-defined acute pancreatitis: interstitial form ("light" or "edematous") and severe, also known as necro-hemorrhagic or "necrotizing" which usually implies some degree of pancreatic necrosis, peripancreatic, or both, and with more complications, such as infection of necrosis, peripancreatic fluid collections, abscesses, pseudocysts and even the failure of multiple organs 1 .

Severe acute pancreatitis (PAG) is characterized by having three or more Ranson criteria score, eight or more points in the Apache II (Acute Physiology and Chronic Health Evaluation II), pancreatic complications or the presence of organic bankruptcy 1 .

**FIGURE 1:**
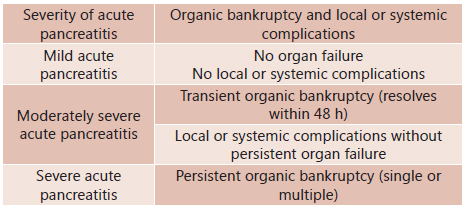
Severity categories according to the Atlanta Criteria Review 3

Adapted from Campos T, heck JG, Assef JC, Rizoli S, B Nascimento, Fraga GP. Severity Ratings in acute pancreatitis. Rev Col Bras Cir. [serial on the Internet] 2013; 40 (2). Available at URL: http: // www.scielo.br/rcbc

However, some authors have suggested revising the Atlanta criteria, proposing the concept of the addition of "moderately severe acute pancreatitis", which includes patients with PAG, but without organ failure ( Figure 1 )^3^.

The aim of this study is to present non systematic review of the PAG, mainly predictive of poor prognosis scores and updates.

## METHOD

Non systematic review of the literature through the evaluation of 28 papers, with emphasis on 13 articles published in indexed journals in the period from 2008 to 2013 in the Lilacs, Medline and Pubmed databases using the following headings: acute necrotizing pancreatitis, prognosis, severity index disease

## RESULTS

### Gravity prognostic factors 

Various clinical, laboratory and image findings have been identified that can predict the PAG development. Obesity is one of the most important negative prognostic factors, and suggested that it increases the risk for both local and systemic complications. Numerous studies have been conducted in order to assess how obesity predicts poor prognosis, and was found that it is an isolated risk factor for PAG. According to Lowenfels et al (2011) obese people have a higher risk of mortality associated with local and systemic complications (OR: 2.9)^5^ ^14^ ^21^ .

Alcohol is another risk factor, as it decreases the threshold for trypsinogen activation, and cause direct toxicity in the acinar and ductal cells causing necrosis 14 .

Old age negatively influences the evolution of the disease, since there is comorbidities increase of over time. Gardner et al (2008) conducted a study in which two groups were divided as follows: patients aged over 70, and less than 70. On first, the mortality was 21.4% and in the later the same ratio reached 7.1% (OR=3, p=0.028). Other studies, such as Lindkvist et al also suggested that old age is one of the factors that influence the prognosis of acute pancreatitis (PA) 14 13 .

Moreover, according to Lindkvist et al (2011), the active tobacco use has been suggested as one predisposing factor for PAG through mechanisms not yet well established 13 .

In preliminary studies of genetic susceptibility factors it was established that the presence of polymorphisms in a single potent chemokine gene - known as monocyte chemotactic protein (MCP-1) -, at -2518 A/G position determines that the inflammatory response to PA be systemic and associated with increased mortality 22 .

The hematocrit gets highlighted as a predictor of severity in PA. Values ​​on admission above 44% configure itself as an independent risk for necrosis 26 , while its normality has negative predictive value for PAG greater than 95% 25 . 

As the hematocrit, serum urea is related to the severity of the PA, establishing itself as an independent predictor of mortality. Its dosages above 20 mg/dl, at admission, are associated strongly with higher risks of death, as well as, any increase in value after the start of monitoring 27 . High values ​​of this marker at admission also is associated with prolonged stay at intensive care unit 14 27 .

Serum creatinine, however, still needs more investigation as severity predictor in PAG. There are studies that contradict the hypothesis that its high levels are associated with higher chances of developing pancreatic necrosis 10 .

In addition, a variety of cytokines, chemokines and other inflammatory response markers have been evaluated as PAG predictors as well as the development of multiple organ failure and systems.

The first reports of the PAG correlation with inflammatory cytokines were with the demonstration of increased levels of IL-6 and IL-8 in patients with PAG and thereafter increased levels of IL-1, and currently it is considered that the main mediators are : IL-1, IL-6, IL-8, IL-10, IL-12, IL-18 and TNF-alfa 21 .

IL-6 is cytokine released by macrophages in response to tissue injury. So, it is elevated in severe pancreatitis and pancreatitis chronic process. This justifies the fact that the increase in IL-6 levels is isolated predictive factor of mortality and length of hospital stay, with sensitivity of 89% to 100% and accuracy of 90% in the first 24 h. Some authors consider that the evaluation of IL-6 levels in the first 24 h is much more useful in terms of prognosis than the Ranson and Apache-II score systems 21.

IL-1 proinflammatory cytokine is of great importance in assessing the gravity of the PA, as it is associated with systemic inflammatory response syndrome, because it leads to activation of the coagulation cascade, with microthrombi and dysfunction of endothelial cells, which lose the ability to regulate blood flow. Furthermore, it has been shown that it is the main cytokine involved in systemic and local tissue destruction, and it is the main inflammatory mediator in sterile necrotizing PA. It has been used as a biomarker of disease severity, with a similar accuracy of IL-6 in predicting severe PA during admission: 82% IL-1 vs 88% IL-6 21 .

TNF-alpha cytokine is expressed in the acinar cells which acts as a regulator of other pro-inflammatory mediators and leukocyte adhesion molecules (which act as activators of immune cells). Due to its rapid clearance, is less used as prognostic marker even playing important role in PAG 21 .

The procalcitonin (PCT) is an acute phase reactant recognized as sepsis marker since 1993, when studies showed correlation of its concentration with the severity of inflammation. Since then, its value has been extrapolated for the evaluation of severity in other clinical conditions 16 . In this sense it has been investigated extensively as an early marker of infectious complications in PA 9 . Was also observed that the concentrations of PCT are higher in patients with infected necrosis, and has significant relation in cases of sterile necrosis. KylänpääBäck et al (2006) used a semiquantitative stick test in 162 patients with PA, 38 of which had the severe form. Twenty-four hours after admission the test had VPN of 97% to identify (delete) those patients who later will develop multiple organ failure (cutting point: 0.5 pg/l), with 92% sensitivity and 84 % specificity, showing that higher PCT concentrations reflect more severe systemic infection 15 . Mofidi et al (2009) reported that in patients with PAG, PCT serum levels can distinguish those who will develop infected pancreatic necrosis from those with sterile pancreatic necrosis, although this is not universally accepted. Possible reasons for the discrepancy between the aforementioned studies may be related to the variation of the definition of PAG as well as the variation in treating this condition. However, different causes of acute pancreatitis may affect serum PCT differently; biliary sepsis, for example, have marked influence on its level. Before PCT become widely used in clinical practice, it is required to elucidate with more consistent studies the dosing time and optimal cutoff values ​​that can best predict the progression of pancreatitis to severe grade 18 .

Routine clinical and laboratory data and multifactorial scores, measured on admission and during the first 48 h of hospitalization, are used to estimate the magnitude of the inflammatory response to injury as well as to predict whether or not intensive support will be needed. Hematocrit on admission, C-reactive protein in 48 h, Ranson criteria and Apache II are the most popular. In addition are used Iget -better known as Baltazar criteria -, the Saps II (Simplified Acute Physiology Score II) and prognostic criteria of Glasgow/Imrie, Sofa II, BIsap and Mods 12 . This article, however, will address in more detail the top five most widely used prognostic indexes.

### Biochemical markers

#### PCR (C-reactive protein)

Among the various plasma biochemical markers to estimate severity in PA, PCR continues to be the most useful. Although the maximum serum concentration is achieved after 72 h, it is able to differentiate severe cases of mild cases of PA within the first 24 h with higher sensitivity and specificity, as 80%. According to the UK guidelines for the management of the PA and the Working Group of the Bangkok World Congress of Gastroenterology in 2002, PCR ≥15 mg/dl is adopted as a prognostic factor 6 19 .

### Scores (criteria) of severity 

#### Ranson

**FIGURE 2:**
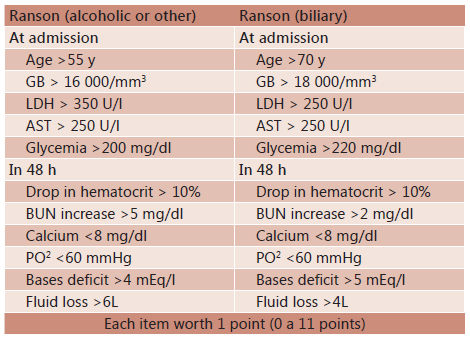
Ranson criteria

Adapted from Feldman M, Friedman L, Brandt L. *Sleisenger and Fordtran's Gastrointestinal and Liver Disease,* Ninth Edition 2010. GB=total leukocytes; LDH=lactate dehydrogenase; AST=aspartate aminotransferase; BUN=blood urea; PO2=partial pressure of oxygen in arterial blood.

Released in 1974 by John HC Ranson, this score was the first widely used in the PA. Initially encompassed 43 clinical and laboratory parameters, and of these, only 11 have shown to be related to mortality and morbidity. Therefore, Ranson criteria were amended in 1982 and currently consist of 11 parameters, of which five are assessed on admission and other during the first 48 h ( Figure 2 )^19^. The presence of three or more criteria within 48 h of admission, classifies as severe pancreatitis. It has a sensitivity of 75% to 87%, specificity of 68% to 77.5%, PPV of 28.6% and 49% and NPV of 91% to 94.5% 25 .

Still, there is another interpretation related to the criteria amount and the probability of mortality, suggesting score in between 0 and 2 related to 2% mortality chance. But score between 3 and 4 increases the chance of death to 15%. And yet, score from 5 to 6 reaches index of 40% mortality chance and 100% when the score is 7 to 8 24.

#### Apache II (1985)

**FIGURE 3:**
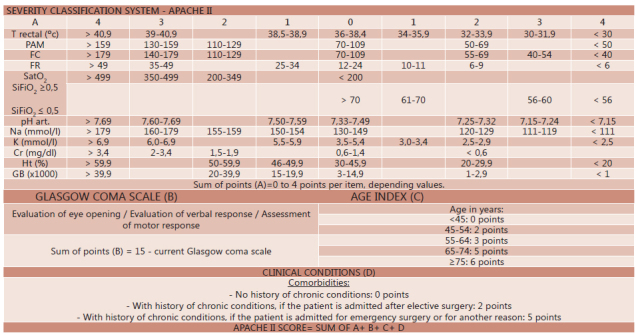
Apache II severity classification system

Adapted from Knaus WA; Draper EA; Wagner DP, Zimmerman JE. Apache II: a severity of disease classification system. Crit care Med 1985; 13(10): 818-29. T rectal=rectal temperature; MAP=mean arterial pressure; HR=heart rate; RR=respiratory rate; SpO2=oxygen saturation; pH art=arterial pH; Na=plasma sodium; K=plasma potassium; Cr=creatinine; Ht=hematocrit; GB=total leukocyte

It is still one of the more used ways for PA severity stratification and risk of mortality 8 . It has 12 evaluation parameters, and extra score based on age and the presence of chronic disease ( Figure 3 ) 14 25 . It has sensitivity of 76% and specificity of 61.5% to assess the PA severity PA 28 . Atlanta classification considers the diagnosis of severe PA when, by Apache classification, are assigned eight or more points^1^ . It has the advantages of being able to be calculated within the first 24 h after patient's admission to hospital and can be performed daily in the evaluation of patient outcomes. The addition of BMI in Apache II score - known as Apache-O - adds one point to BMI of >25-30 kg/m^2^ and two points to BMI >30 kg/m^2^. Johnson reported that this system improves severe pancreatitis forecast 7 .

#### Saps II

**FIGURE 4:**
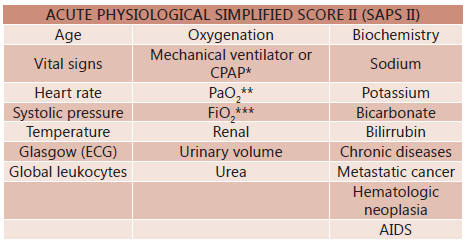
Acute physiological simplified score II (Saps II)2

Adapted from Bouch DC, Thompson JP. *Severity scoring systems in the critically ill* . Continuing Education in Anaesthesia, Critical Care & Pain. 2008; 8(5): 181-185. *CPAP=continuous positive airway pressure; ** PaO 2=partial pressure of oxygen in arterial blood; *** FiO 2=fraction of inspired oxygen

The Simplified Acute Physiology Score (Saps) model was developed in France by Le Gall et al, in 1983, changed to Saps II in 1993. It is an alternative version of the Apache scale, and was originally released shortly after this and subsequently updated to its second version. However, this tool is used most often in the intensive care unit compared with the Apache. It has sensitivity and specificity respectively 87.5% and 77.8% for predicting mortality. From this, obtains the PPV of 18.2% and 99.1% respectively. Thus, the Saps II should be applied in the first 24 h of admission in intensive care unit and consists of 12 immediate variables, while also taking into consideration the age and comorbidities acquired before admission ( Figure 4 ). The cut-off used is ≥34 12 2 . To help the scale application and estimate the risk of patient mortality, can be used online public domain programs, found at websites such as: http://clincalc.com/IcuMortality/SAPSII.aspx?example .

Some authors have suggested moderate accuracy of Saps II in prognostic evaluation of the PA. Thus, the parallel association of Iget, when the cutting score Saps II is reached, is able to increase the accuracy of predicting severe grade of PA. According to Balthazar, the CT scan done after 48-72 h of the onset of symptoms has greater diagnostic accuracy.

### ACUTE PHYSIOLOGICAL SIMPLIFIED SCORE II (SAPS II)

#### Iget

**FIGURE 5:**
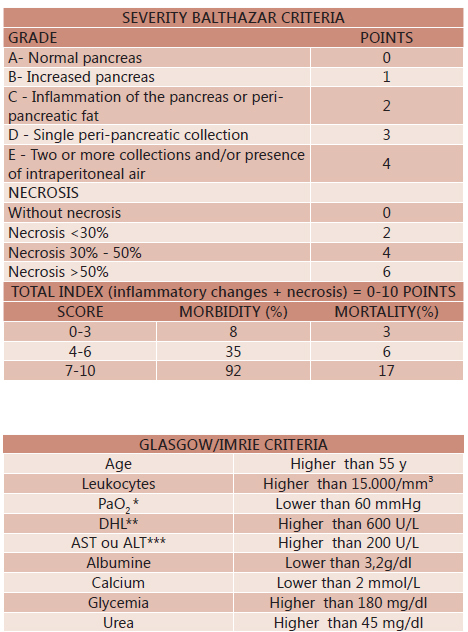
Severity Balthazar 4and Glasgow (Imrie) criteria for acute pancreatitis 11

Adapted from Delrue L. J; Waele J. J; Duyck P. O. *Acute pancreatitis: radiologic scores in predicting severity and outcome* . 2010; 35(3):349-61

Adapted from de Ledesma-Heyer JP, Amaral JA.*Pancreatitis aguda* . Medicina Interna de México. (2009; 25(4): 285-294). *PaO 2=partial pressure of oxygen in arterial blood; **DHL=lactate dehydrogenase; ***AST=aspartate aminotransferase; ALT=alanine aminotransferase

In 1985 Balthazar et al introduced severity score based on the presence of pancreatic and peri-pancreatic inflammation and fluid collections evidenced by computed tomography, and the PA classified into five grades from A to E. This score was achieved without the need to use contrast, making it impossible to detect pancreatic necrosis, reducing its prognostic value. In this regard, it has become crucial to the introduction of intravenous contrast in the CT scan enabling the detection of pancreatic necrosis, it was graded as zero, 30%, 50% or more than 50%. This new classification (extension pancreatic necrosis) was combined with ideally prepared by Balthazar (grades A to E), yielding the Staging CT Severity Index (Iget) which scores pancreatitis from 0 to 4, according rating of A to E, and the scores 0, 2, 4 or 6 according to the classification of the percentage of the extent of necrosis. The points are added up and the Iget varies between 0 and 10. ​​Iget values ≥7 already represent 92% morbidity and 17% mortality (Figure 5 ). Because the contrast medium used for the detection of pancreatic necrosis increase the risk of nephrotoxicity and could aggravate the course of the PA, the use of Iget is still questionable, not being considered by several authors 14 23 .

#### Glasgow/Imrie prognostic markers 

**FIGURE 6:**
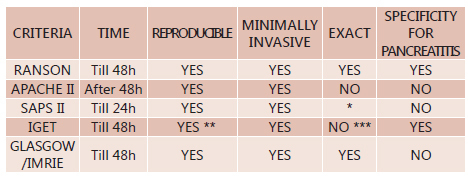
Guide to aid in selecting the prognostic score of multifactorial severity

With sensitivity of 72% and specificity of 84%, the prognostic indicators of Glasgow are used on PAG prediction of both alcoholic and biliar cause 11 . Based on the Ranson score, the scale was proposed by Imrie for the first time in 1984, and seeks to make the linkage between clinical, specific laboratory and radiologic markers of PA, with the severity of the condition and its expected result. Can be calculated at any time within the first 48 h of admission and measures just eight parameters 17 ( Figure 6 ). In the presence of three or more criteria at 48 h, the presence of a severe case of PA is faced with the aforementioned sensitivity and specificity.

Figure 6 shows some criteria to help in choosing the prognostic score of multifactorial severity that can be used.

*Some studies suggest a moderate accuracy of SAPS II concerning the prognosis of acute pancreatitis; ** lower reproducibility for the extension of the definition of pancreatic necrosis has no effect on the calculation of Iget reproducibility; *** tomographic studies show early findings misconceptions related to ischemia of the gland which according to Balthazar, examinations after three days have higher diagnostic accuracy, and staging of acute pancreatitis, CT should only be carried out after 48-72 h from start of clinical condition.

Acute pancreatitis is a disease that has several prognostic factors; they are useful in predicting mortality and the development of the severe form. Some of these factors such as IL-6 and PCR may alone be determinant for clinical evolution to the most severe grade. However, many multifactorial criteria have been widely used in clinical practice. These may have disadvantages, such as the need for more time since the clinical outbreak till its full application, as Ranson, Glasgow and Iget criteria, and the complexity of the evaluation system, such as Apache II and in a lesser degree the Saps II, being the latter the most widely used scores and described in the literature.

## CONCLUSION

Acute pancreatitis is a disease in which employ various prognoses, useful factors in predicting mortality and severe form of development. It is suggested that the association of a multifactor score, especially Saps II associated with Iget, allows increased accuracy in predicting prognosis. However, professional preferences, the service experience as well as the tools available, are factors that have determined the choice of the most appropriate predictor score.
